# Draft Genome Sequence of Pseudomonas sp. Strain RGM 3321, a Phyllosphere Endophyte from Fragaria chiloensis subsp. *chiloensis* f. *patagonica*

**DOI:** 10.1128/mra.00335-22

**Published:** 2022-06-22

**Authors:** Jean Franco Castro, Matías Guerra, Jorge Carrasco-Fernández, Javiera Ortiz-Campos, Diego Cares-Gatica, Carolina Campos-Quiroz, Francisco Correa, M. Francisca Beltrán, Boris Sagredo, Jorge H. Valdés

**Affiliations:** a Instituto de Investigaciones Agropecuarias, INIA-Quilamapu, Chillán, Chile; b Instituto de Investigaciones Agropecuarias, INIA-Rayentué, Rengo, Chile; c Center for Bioinformatics and Integrative Biology, Facultad de Ciencias de la Vida, Universidad Andrés Bello, Santiago, Chile; University of Arizona

## Abstract

Pseudomonas sp. strain RGM 3321 is a phyllosphere endophyte from Fragaria chiloensis subsp. *chiloensis* f. *patagonica* that harbors genes associated with plant growth promotion pathways, as well as genes typically found in plant pathogens.

## ANNOUNCEMENT

Fragaria chiloensis subsp. *chiloensis* f. *patagonica* is the wild form of the Chilean strawberry, which grows in the mountains and in coastal environments (1). In January 2021, a sample of this species was removed from forest soil in the Yungay Precordillera, Ñuble, Chile (−37.05905, −71.64625). The phyllosphere was surface sterilized by submerging the tissues in 70% ethanol (1 min), 1.5% NaClO (3 min), and 96% ethanol (1 min) and finally rinsed three times. The tissues were subsequently ground in physiological solution, and volumes of 100 μL of serial dilutions were inoculated on King's B (KB) agar medium supplemented with 25 μg/mL nystatin and 50 μg/mL cycloheximide. Plates were incubated at 25°C for 48 h. A UV-fluorescent colony was streaked on KB agar and incubated under the same conditions. This step was repeated twice to obtain axenic cultures. The isolate was deposited in the Chilean Collection of Microbial Genetic Resources (CChRGM), under the code RGM 3321. Strain RGM 3321 grown in yeast extract-malt extract-dextrose broth supplemented with 1% l-tryptophan produced 106.47 μg/mL indole acetic acid (IAA) and grown on NBRIP agar displayed a phosphate solubilization index of 2.4, suggesting potential plant growth-promoting traits (2–4).

Two genomic DNA libraries were constructed using the Nextera XT library preparation kit (Illumina, USA) and sequenced on an Illumina HiSeq/NovaSeq platform using a 250-bp paired-end protocol at MicrobesNG (UK). Whole-genome sequencing reads were adapter trimmed using Trimmomatic v0.30 with a sliding window quality cutoff value of Q15 (5, 6). *De novo* assembly was performed using SPAdes v3.7 (7). Contigs were annotated using the NCBI Prokaryotic Genome Annotation Pipeline (PGAP) v6.0 (8). The assembled genome has a size of 6,380,675 nucleotides (nt), distributed in 121 contigs (the largest contig was 609,627 nt), with mean coverage of 82.91×, an *N*_50_ value of 171,869 nt, and a G+C content of 58.41%; 5,669 genes and 56 tRNAs were predicted from the annotation. On the EzBioCloud webserver (9), RGM 3321 shared the greatest 16S rRNA gene similarity with the phytopathogenic species of the Pseudomonas syringae group (10), i.e., Pseudomonas KCTC 12500^T^ (99.93%) and Pseudomonas congelans DSM 14939^T^ (99.86%) and Pseudomonas cerasi 58^T^ (99.86%).

BLAST searches were performed using SequenceServer v2.0.0 (11). Strain RGM 3321 harbors one copy of a gene for 1-aminocyclopropane-1-carboxylate (ACC) deaminase (*acdS*), which is involved in the degradation of ACC, a precursor of ethylene in plants (12, 13). The strain contains genes for a quinoprotein glucose dehydrogenase (*gcd*) and the pyrroloquinoline quinone (*pqq*) operon (14), suggesting a mechanism for phosphate solubilization in soil via gluconic acid synthesis (15, 16). The identification of *iaaM* and *iaaH* genes suggests production of IAA via the indole-3-acetamide (IAM) and/or indole-3-acetonitrile (IAN) pathways (17, 18). Genes encoding a nitrile hydratase (*nthAB*) were also found, suggesting the transformation of IAN into an IAM intermediate in the IAN pathway (19; Table 1).

**TABLE 1:** Genome sequence features of Pseudomonas sp. strain RGM 3321

Trait and protein name (gene name) or BGC	RGM 3321 protein code	Identity to reference protein (%)	UniProt accession no. for reference protein	Strain encoding the BGC	Similarity to reference BGC (%)^*a*^
Trait and protein name					
Phosphate solubilization					
Quinoprotein glucose dehydrogenase (*gcd*)	RGM3321_17085	69.27	A0A0B6F0P5		
Coenzyme PQQ synthesis protein A (*pqqA*)	RGM3321_09145	95.83	Q3K5R0		
Coenzyme PQQ synthesis protein B (*pqqB*)	RGM3321_09140	91.42	C3K348		
Pyrroloquinoline-quinone synthase (*pqqC*)	RGM3321_09135	92.83	Q88QV6		
PqqA binding protein (*pqqD*)	RGM3321_09130	68.97	Q4K4U9		
PqqA peptide cyclase (*pqqE*)	RGM3321_09125	89.10	Q4K4U8		
Coenzyme PQQ synthesis protein F (*pqqF*)	RGM3321_09150	44.84	P55174		
IAA production					
Tryptophan 2-monooxygenase (*iaaM*); IAM pathway	RGM3321_01470	94.61	P06617		
Indoleacetamide hydrolase (*iaaH*); IAM pathway	RGM3321_01475	93.02	P06618		
Indole-3-pyruvate decarboxylase (*ipdC*); indole-3-pyruvate pathway	RGM3321_13685	26.95	A0A5E6Q147		
Nitrilase (*nit*); IAN pathway	RGM3321_22075	28.52	K9NKH3		
Aldehyde dehydrogenase family protein (*aldA*); IAN pathway	RGM3321_15565	94.16	Q88BC5		
Aldehyde dehydrogenase family protein (*aldB*); IAN pathway	RGM3321_11565	97.57	Q88BC5		
Aldehyde dehydrogenase family protein (*aldB*); IAN pathway	RGM3321_06435	45.50	Q88BC5		
ACC deaminase activity					
ACC deaminase (*acdS*)	RGM3321_08860	88.76	Q51813		
Leucine-responsive regulatory protein (*acdR*)	RGM3321_08855	80.47	K9NP20		
T3SS					
Hypersensitivity response secretion protein HrpJ (*hrpJ*)	RGM3321_26170	98.77	Q05395		
Lipoprotein (*hrcJ*)	RGM3321_26075	90.30	G3XDC8		
Type III secretion protein HrcR (*hrcR*)	RGM3321_26130	93.00	Q887B8		
Type III secretion protein HrcS (hrcS)	RGM3321_26125	94.32	G3XDB8		
Type III secretion protein HrcT (*hrcT*)	RGM3321_26120	84.09	G3XDD0		
Type III secretion protein HrcU (*hrcU*)	RGM3321_26115	85.52	Q887B9		
Hypersensitivity response secretion protein HrpI (*hrpI*)	RGM3321_26165	98.99	P35655		
T3SS ATPase SctN (*sctN*)	RGM3321_26155	99.33	Q52371		
V-type ATP synthase subunit E (*hrpE*)	RGM3321_26085	76.17	Q887C4		
Type III secretion protein HrcQb (*hrcQb*)	RGM3321_26135	93.98	Q60235		
T3SS regulator					
RNA polymerase sigma factor HrpL (*hrpL*)	RGM3321_26175	95.70	P37929		
Hrp pilus protein HrpA1 (*hrpA*)	RGM3321_26060	99.07	Q52420		
T3SS key effector					
Type III effector HopAA1 (*hopAA1*)	RGM3321_26015	74.74	G3XDB9		
Type III effector AvrE1 (*avrE1*)	RGM3321_26040	67.83	Q887C9		
Type III effector HopM1 (*hopM1*)	RGM3321_26030	93.87	Q4ZX82		
BGC					
Pyoverdine				Pseudomonas protegens Pf-5	22
Syringafactin				Pseudomonas syringae pv. tomato DC3000	66
Syringolin A				Pseudomonas syringae pv. syringae B301 D-R	100
Syringomycin				Pseudomonas syringae pv. syringae B728a	100

a Similarity of the BGC found in the genome of RGM 3321 to the reference BGC used by antiSMASH.

Fourteen biosynthetic gene clusters (BGCs) were predicted from the RGM 3321 genome using antiSMASH 6.0 (20), four of which, including the pyoverdine (21), syringomycin (22), syringolin A (23), and syringafactin (24) BGCs, are present in plant-pathogenic bacteria (Table 1). In addition, RGM 3321 contains a type III secretion system (T3SS) gene and additional auxiliary genes (25), including an homolog of the RNA polymerase sigma factor (*hrpL*), which is a master regulator of the T3SS that interacts with a conserved *hrp* box motif and promotes the expression of effectors and other virulence factors (26), including HopAA1, which specifically enhances the epiphytic bacterial survival/growth in plants (27).

The draft genome of Pseudomonas sp. strain RGM 3321 expands our knowledge about the bacterial diversity of Chilean wild plants, revealing plant growth-promoting genes and additional open reading frames associated with plant virulence factors. All tools were run with default parameters unless otherwise specified.

### Data availability.

This whole-genome shotgun project has been deposited at DDBJ/ENA/GenBank under the accession number JALHBH000000000. The version described in this paper is version JALHBH000000000.1. The raw data are available under SRA accession numbers SRR18554678, SRR18554679, and SRR18554680. All project data are available under BioProject accession number PRJNA820724.
